# Supplementary material for: Bradykinin and Neurotensin Analogues as Potential Compounds in Colon Cancer Therapy
Source: Int J Mol Sci. 2023 Jun 1;24(11):9644. doi: 10.3390/ijms24119644 (PMC10253536; doi:10.3390/ijms24119644)
Supplement: Supplementary file 1 [file ijms-24-09644-s001.zip › ijms-2349015-supplementary.pdf]

Analitical data of the designed peptides

Characterization of the peptides

Peptides were purified using a preparative reversed-phase high-performance liquid chromatography system (RP-HPLC) (Shimadzu, Kyoto, Japan) with a Jupiter Proteo column (4 μM, 90 Å, 250 × 10 mm); (Phenomenex, Torrance, CA , USA). The purity (> 95%) of the peptides was determined using an analytical RP-HPLC system (Shimadzu, Kyoto, Japan) with Jupiter Proteo column (4 μM, 90 Å; 250 × 4.6 mm), (Phenomenex, Torrance, CA , USA) and the linear gradient of solution B in A from 5% to 95% over 30 minutes with a flow rate of 1 mL/min. The eluents used were: A - 0.1% aqueous solution of TFA and B - 80% solution of acetonitrile in aqueous 0.1% TFA (v/v). The mass spectra of the peptides were recorded using a Bruker BIFLEX III and autoflex maX MALDI TOF mass spectrometers or for NT-14 only, by mass spectrometry with an ESI LCMS IT TOF device (Shimadzu, Kyoto, Japan). A linear gradient solution B was applied as a mobile phase.

A.

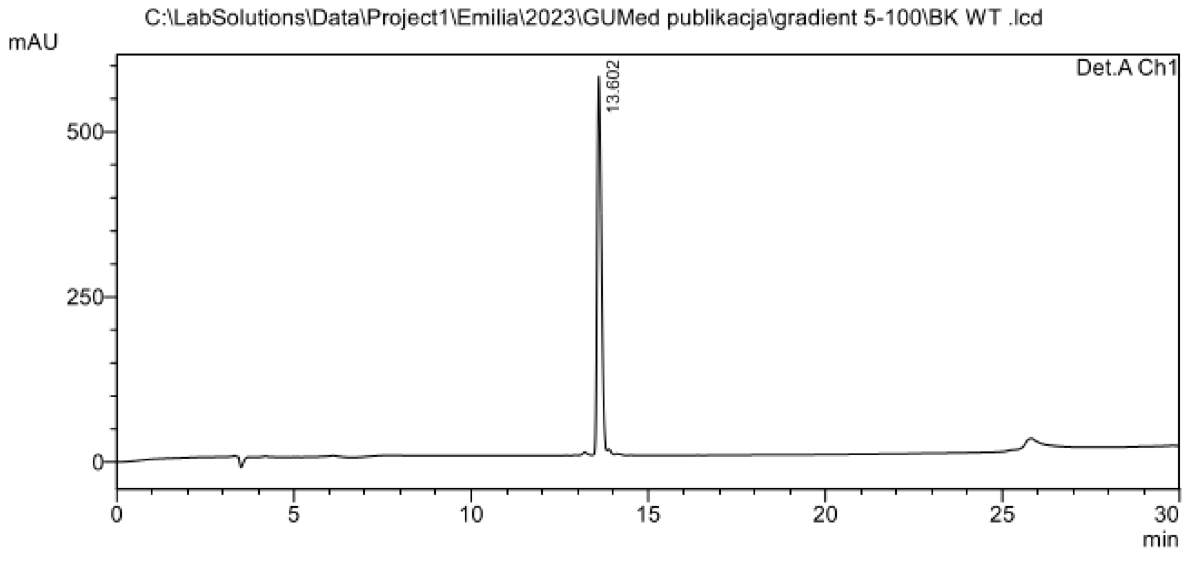

B.

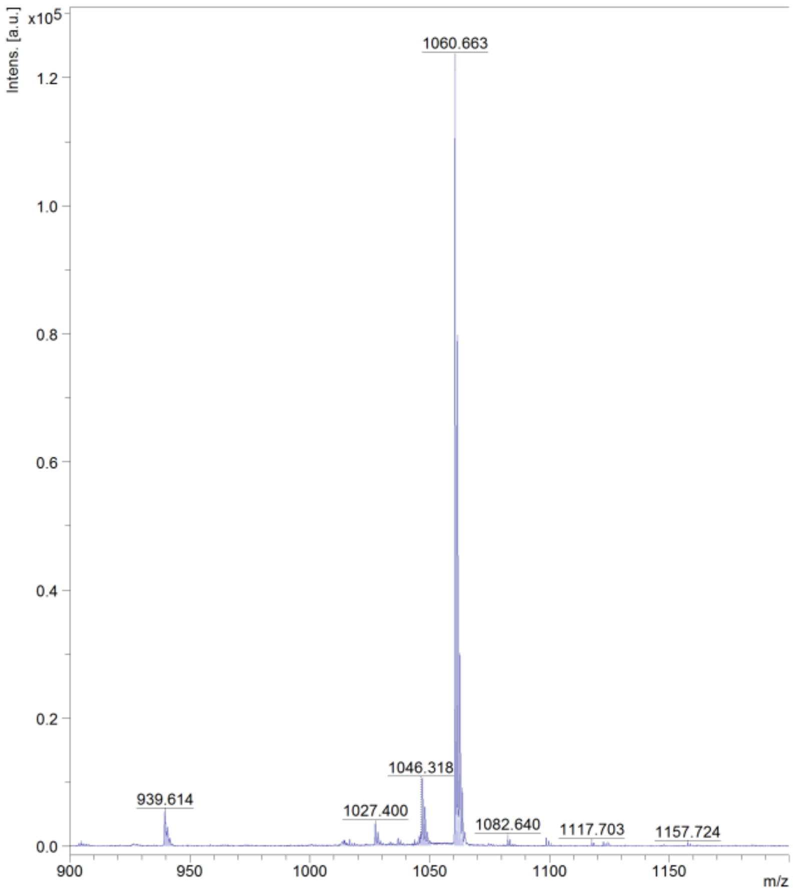

Figure S1. (**A**) Chromatogram RP-HPLC with the tR 13.60 min and (**B**) mass spectra of the BK peptide. Calculated mass: 1060.22; found 1060.66.

A.

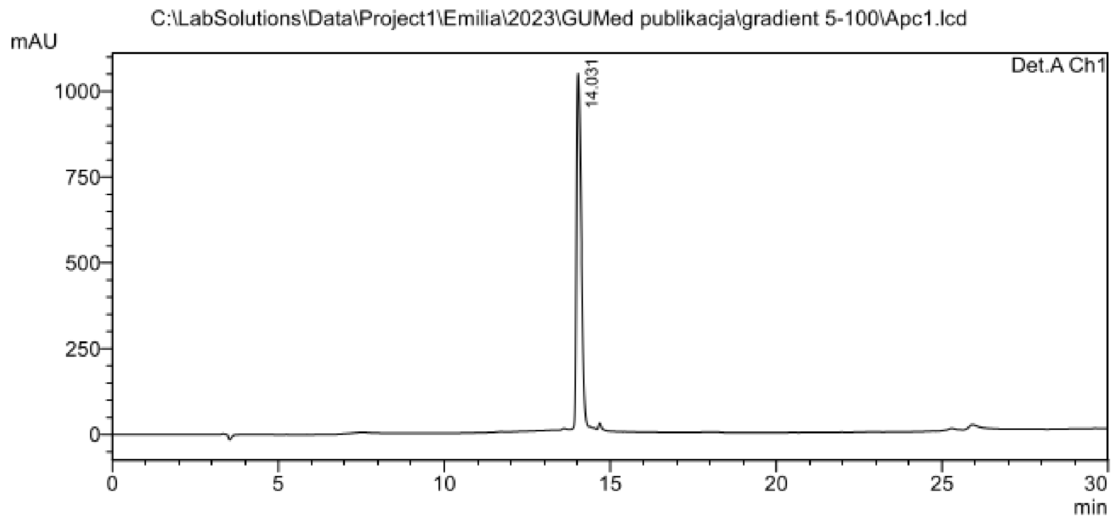

B.

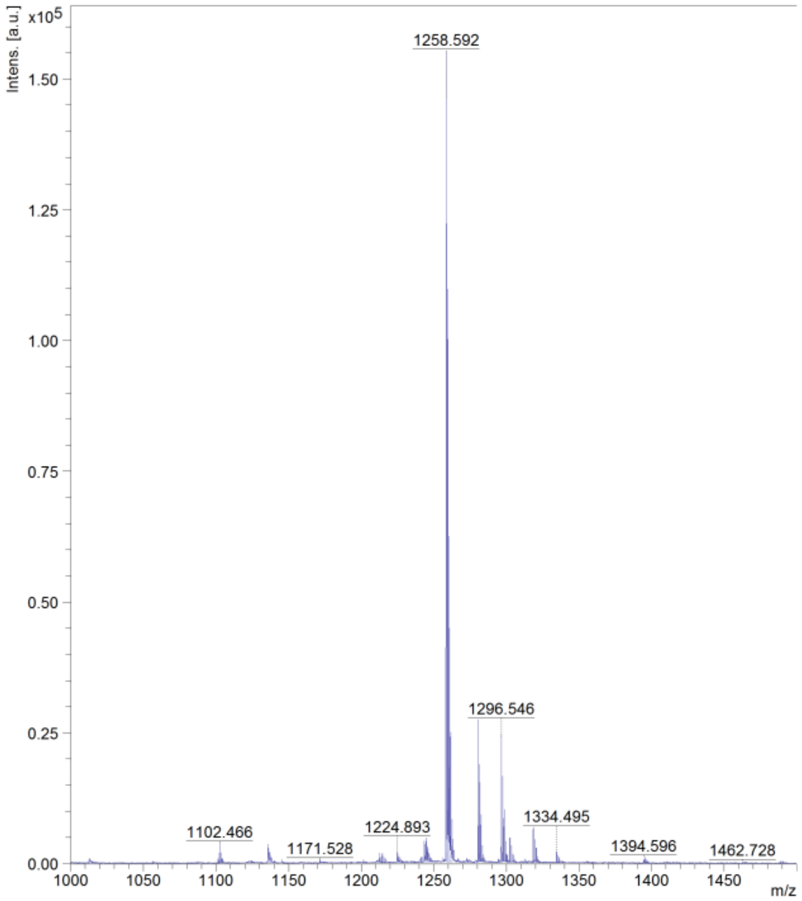

Figure S2. (A) Chromatogram RP-HPLC with the tR 14.03 min and (B) mass spectra of the BK-1 peptide. Calculated mass: 1257.59; found 1258.59 [M+H].

A.

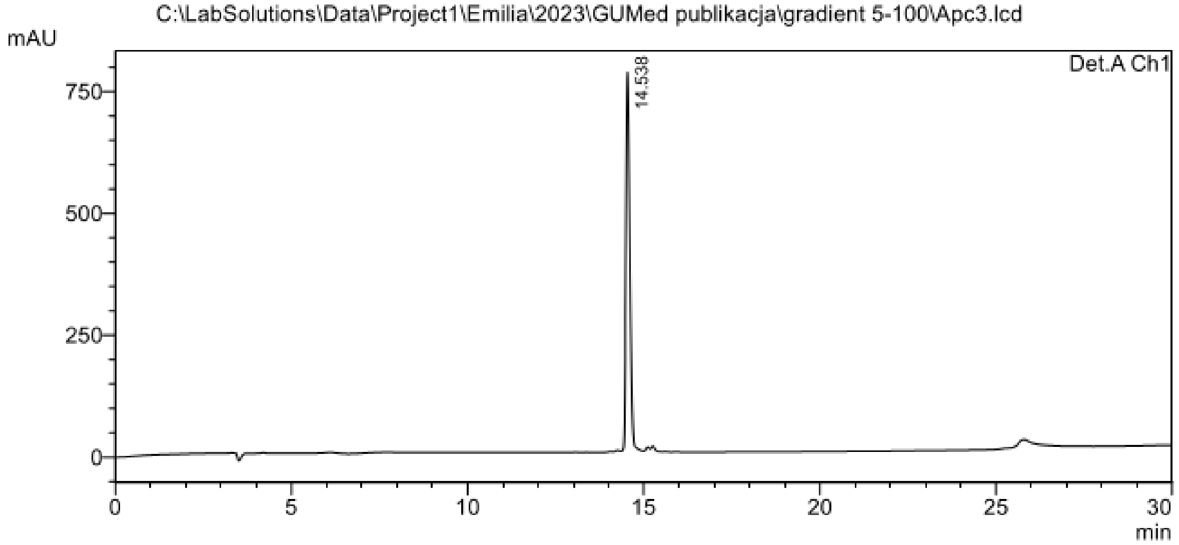

B.

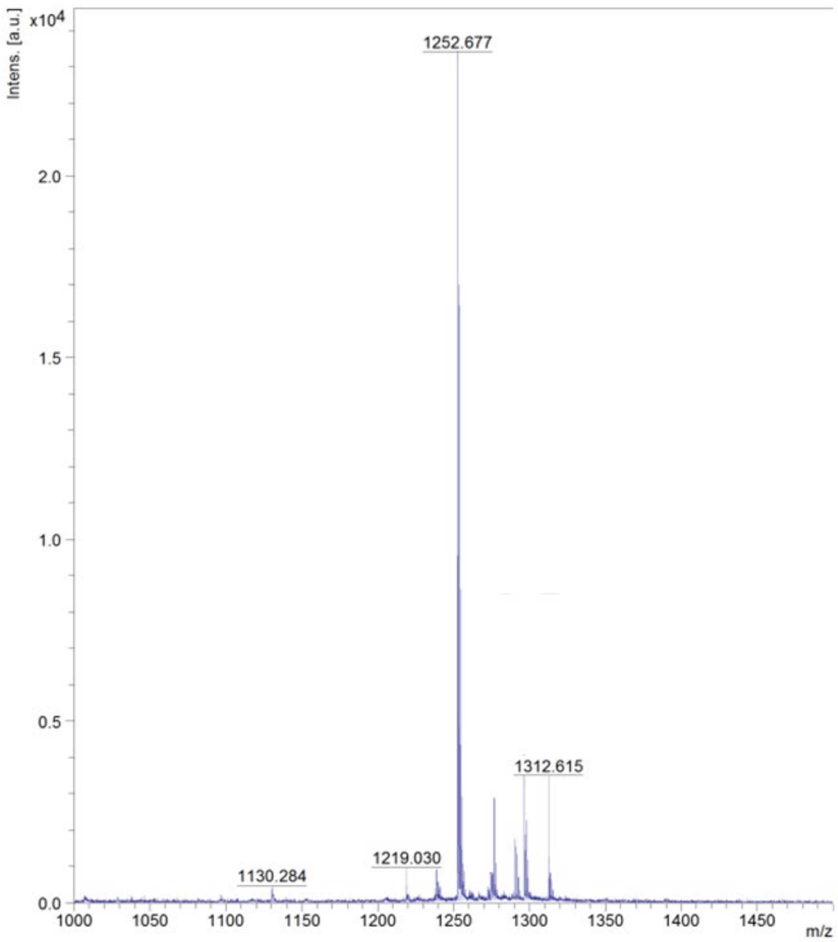

Figure S3. (A) Chromatogram RP-HPLC with the tR 14.53 min and (B) mass spectra of the BK-2 peptide. Calculated mass: 1252.47; found 1252.68.

A.

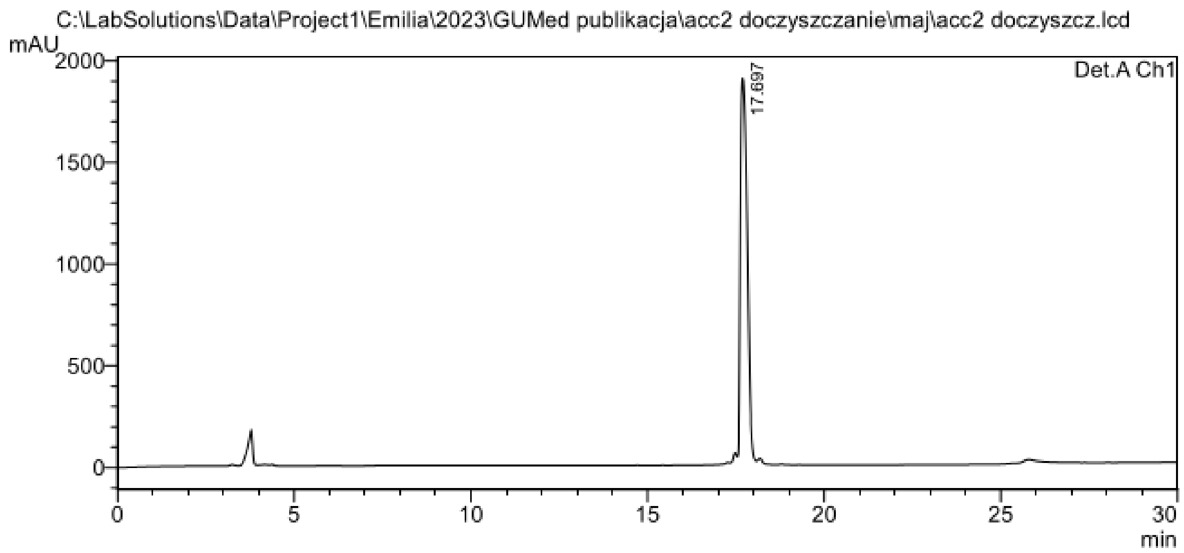

B.

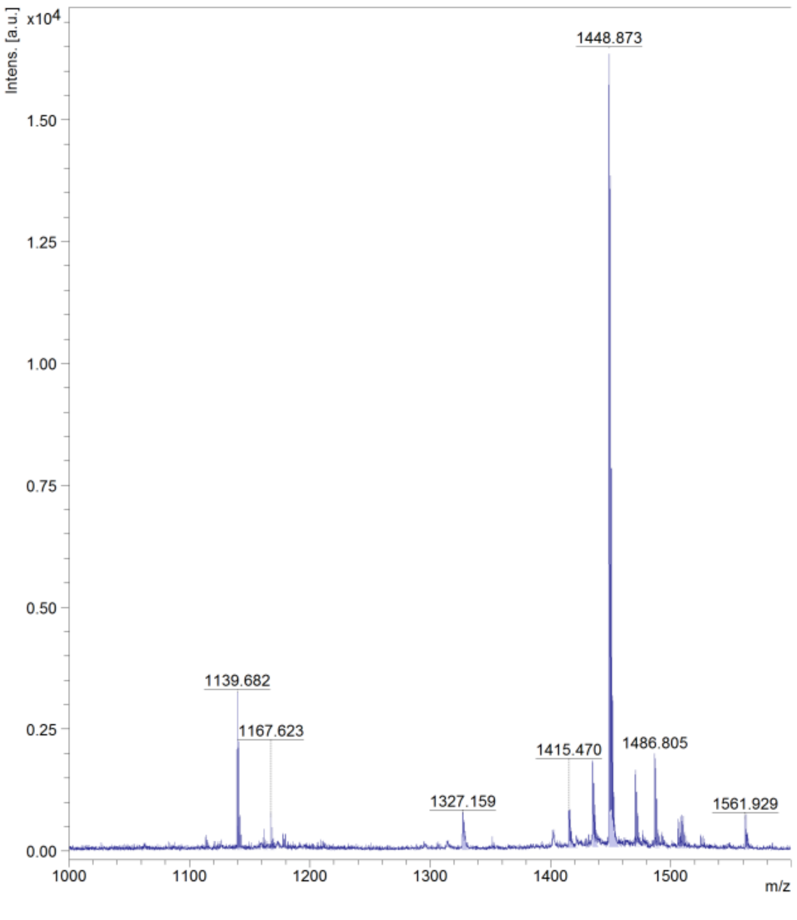

Figure S4. (A) Chromatogram RP-HPLC with the tR 17.69 min and (B) mass spectra of the BK-3 peptide. Calculated mass: 1448.27; found 1448.87.

A.

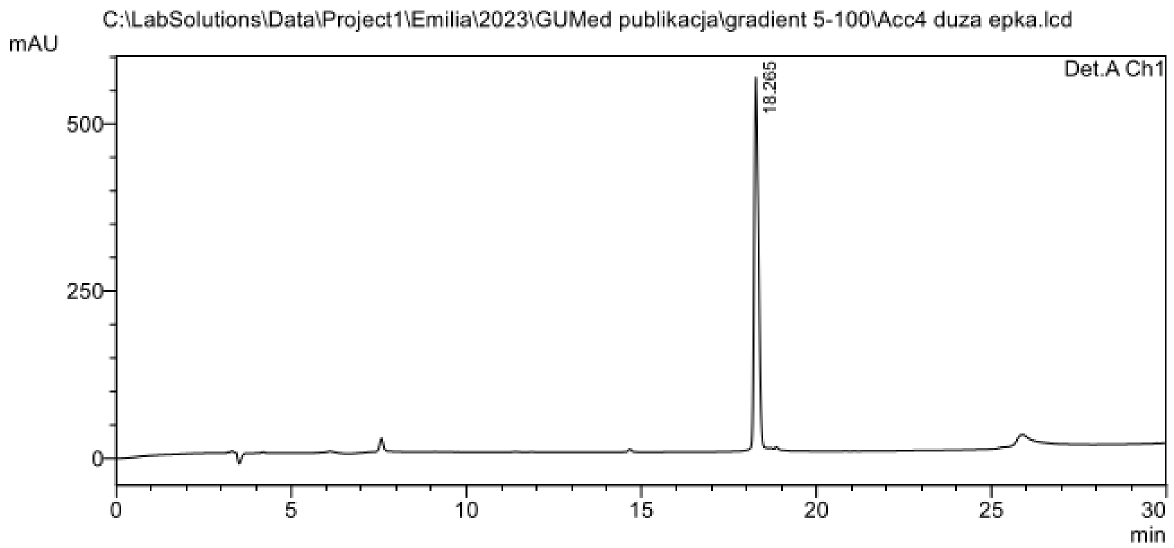

B.

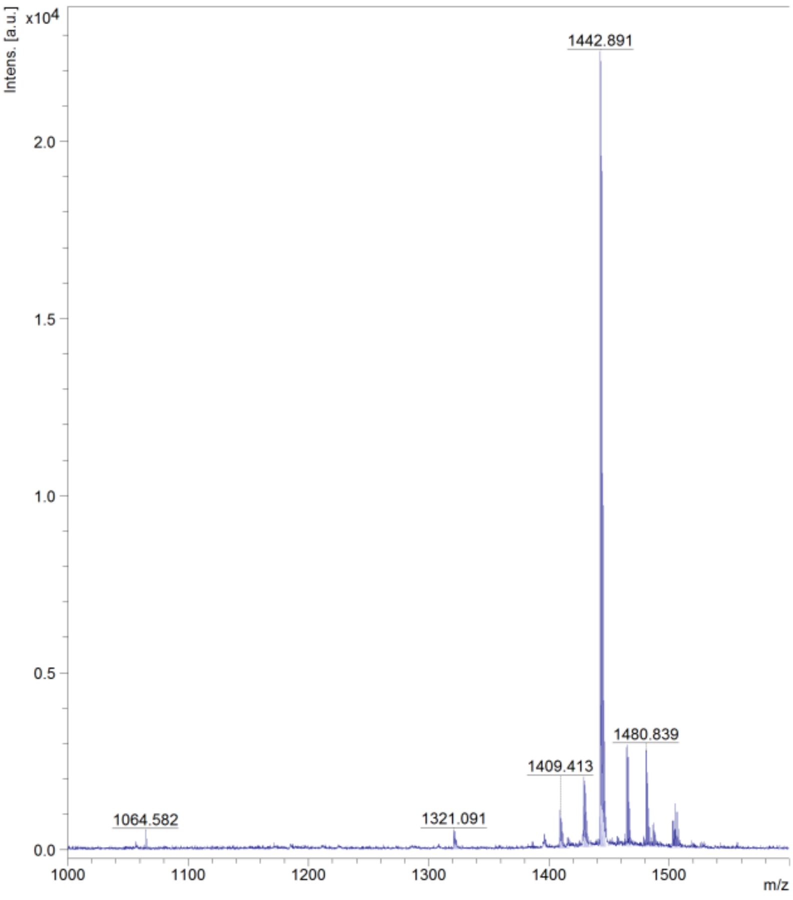

Figure S5. (A) Chromatogram RP-HPLC with the tR 18.26 min and (B) mass spectra of the BK-4 peptide. Calculated mass: 1442.27; found 1442.89.

A.

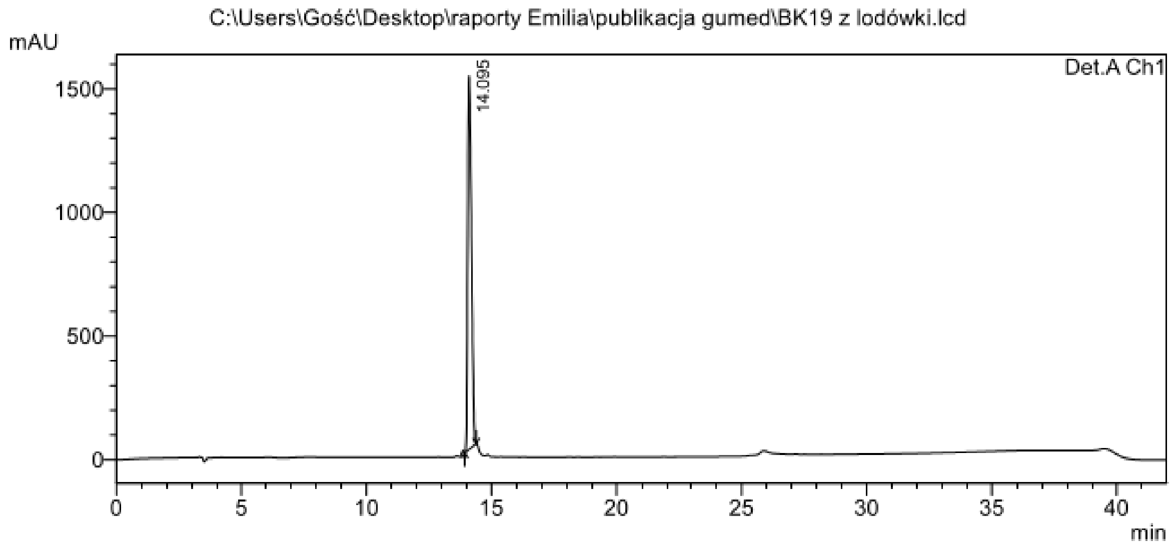

B.

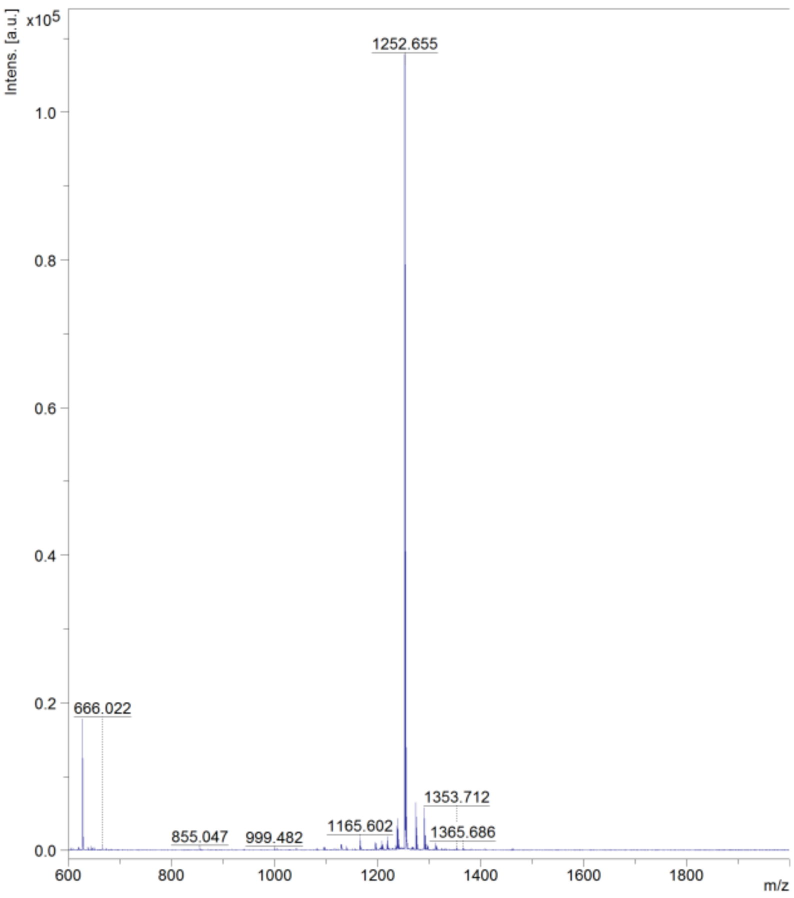

Figure S6. (A) Chromatogram RP-HPLC with the tR 14.09 min and (B) mass spectra of the BK-5 peptide. Calculated mass: 1252.45; found 1252.65.

A.

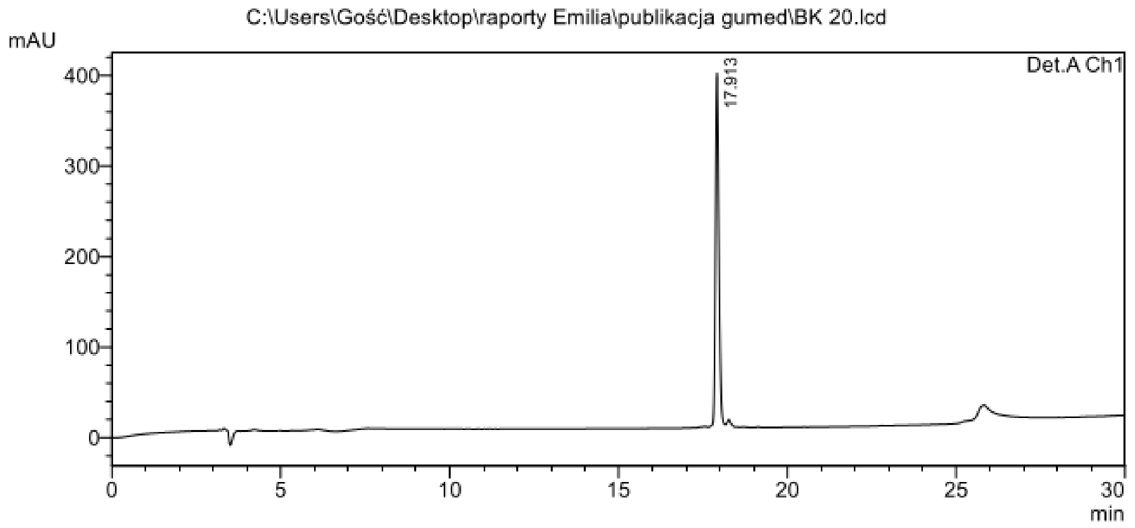

B.

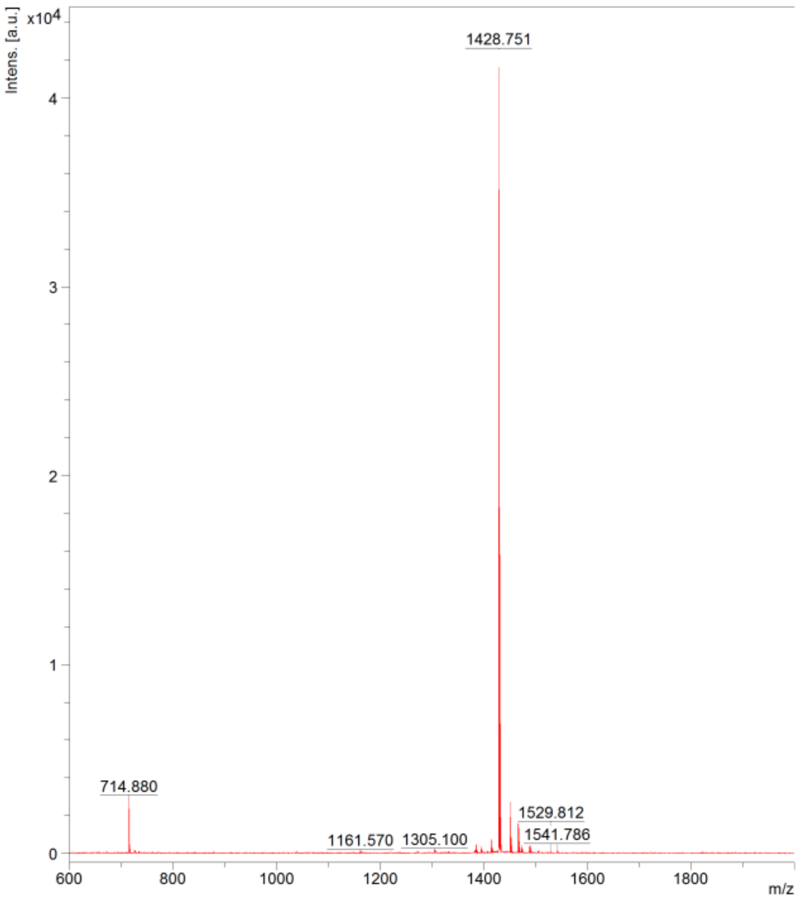

Figure S7. (A) Chromatogram RP-HPLC with the tR 17.91 min and (B) mass spectra of the BK-6 peptide. Calculated mass: 1428.45; found 1428.75.

A.

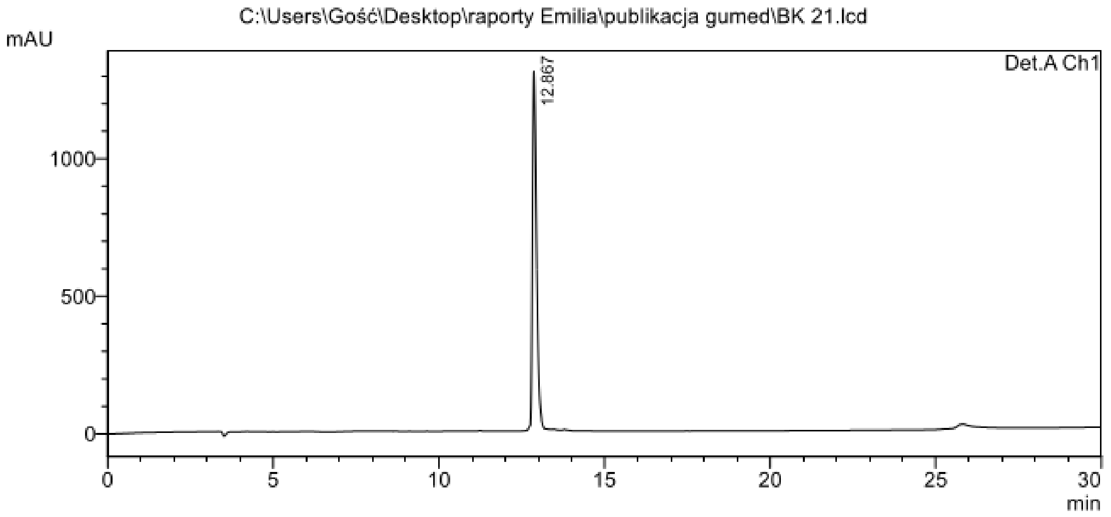

B.

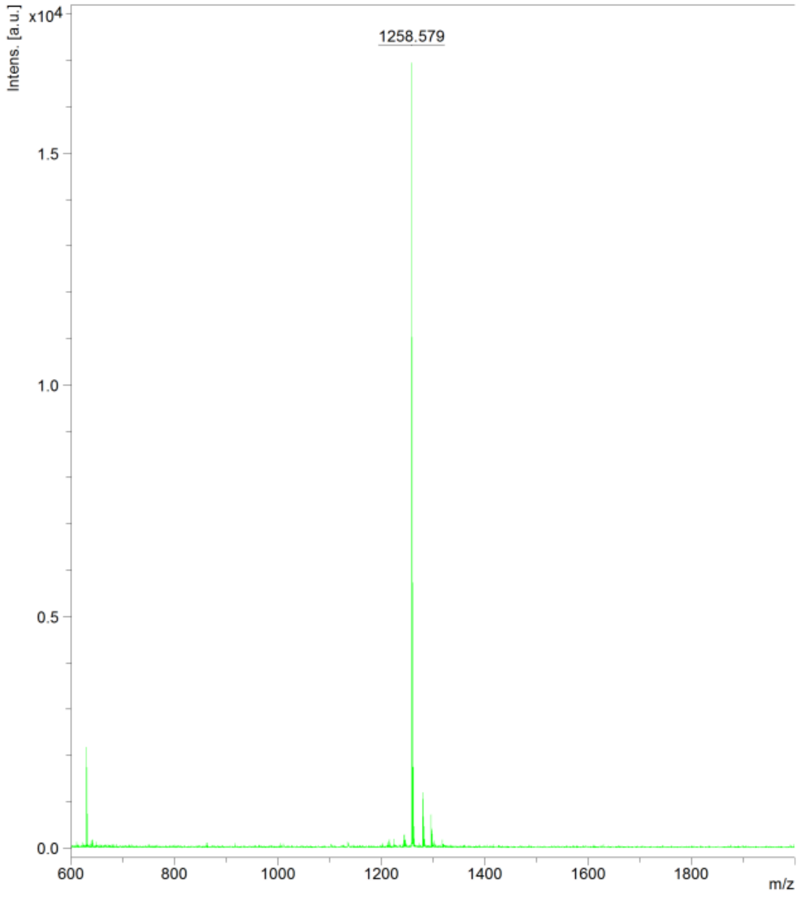

Figure S8. (A) Chromatogram RP-HPLC with the tR 12.87 min and (B) mass spectra of the BK-7 peptide. Calculated mass: 1258.57; found 1258.58.

A.

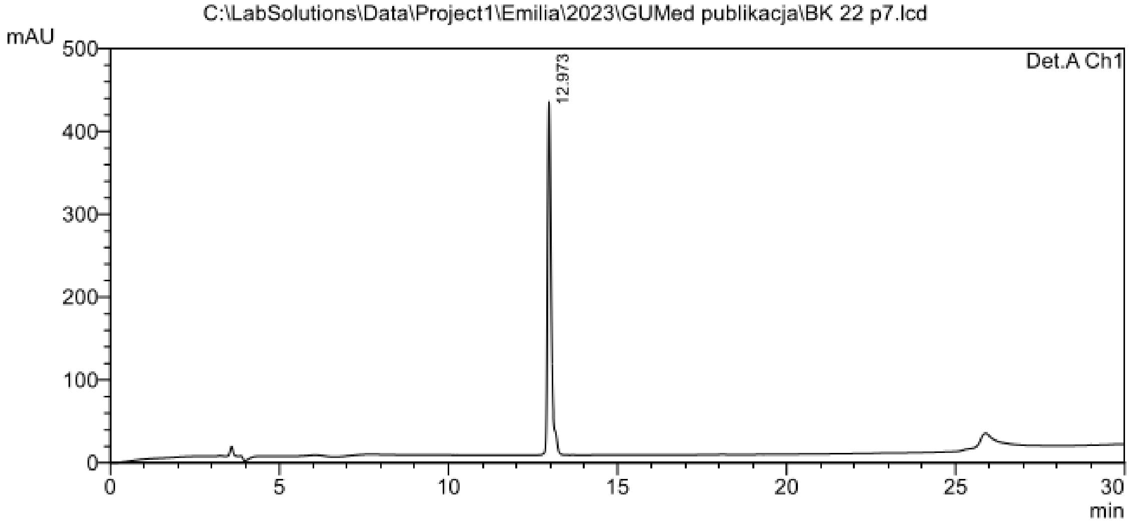

B.

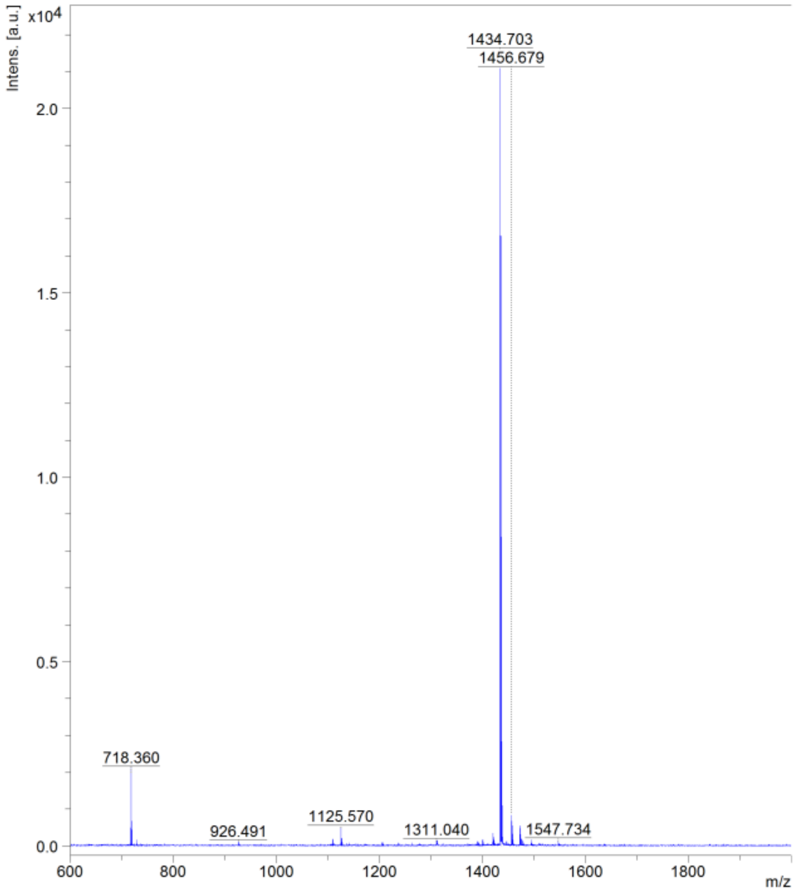

Figure S9. (A) Chromatogram RP-HPLC with the tR 12.97 min and (B) mass spectra of the BK-8 peptide. Calculated mass: 1434.56; found 1434.70.

A.

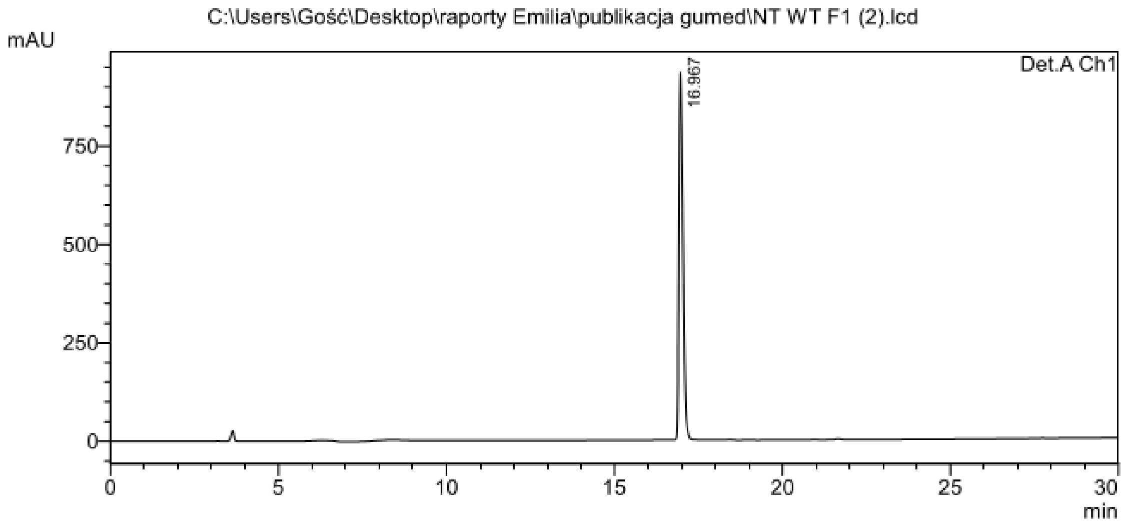

B.

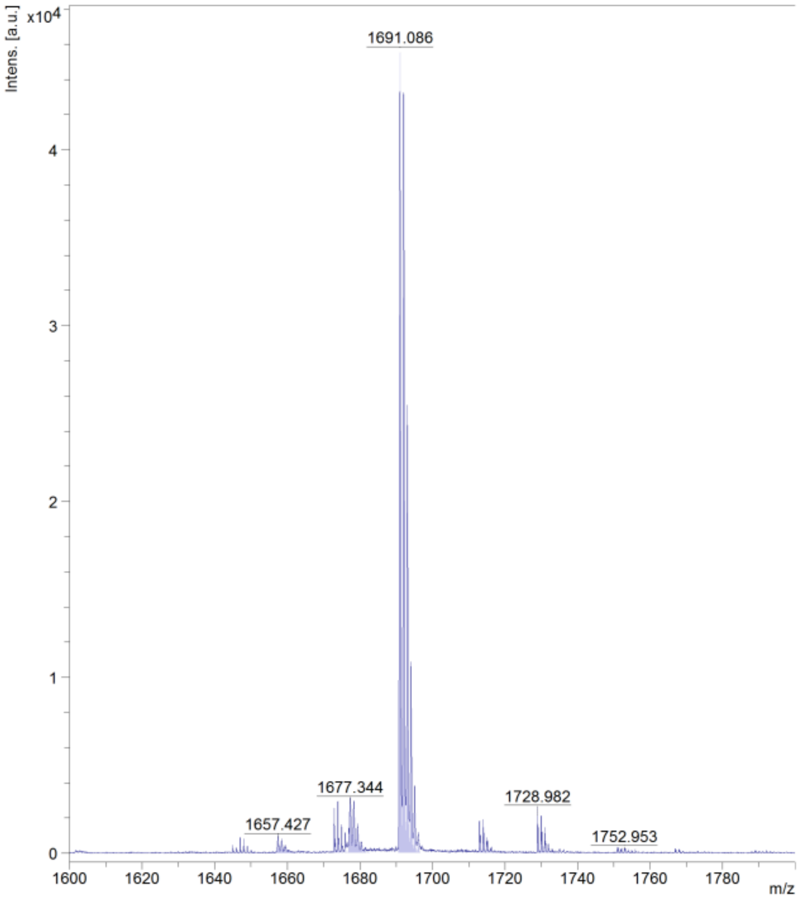

Figure. S10. (A) Chromatogram RP-HPLC with the tR 16.96 min and (B) mass spectra of the NT peptide. Calculated mass: 1690.23; found 1691.08.

A.

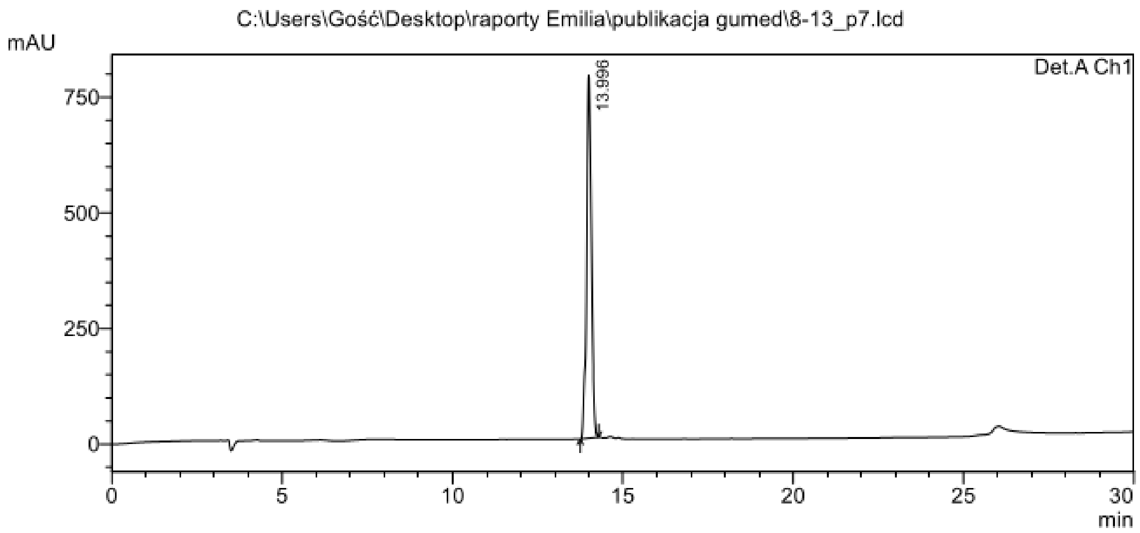

B.

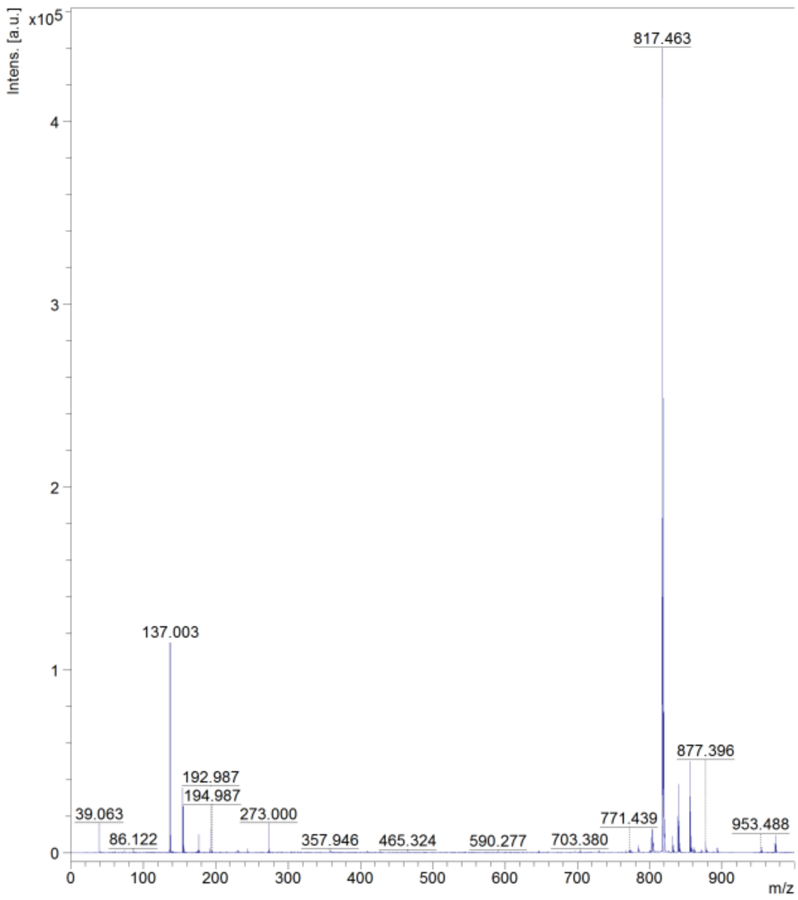

Figure. S11. (A) Chromatogram RP-HPLC with the tR 13.99 min and (B) mass spectra of the NT-9 peptide. Calculated mass: 816.99; found 817,46.

A.

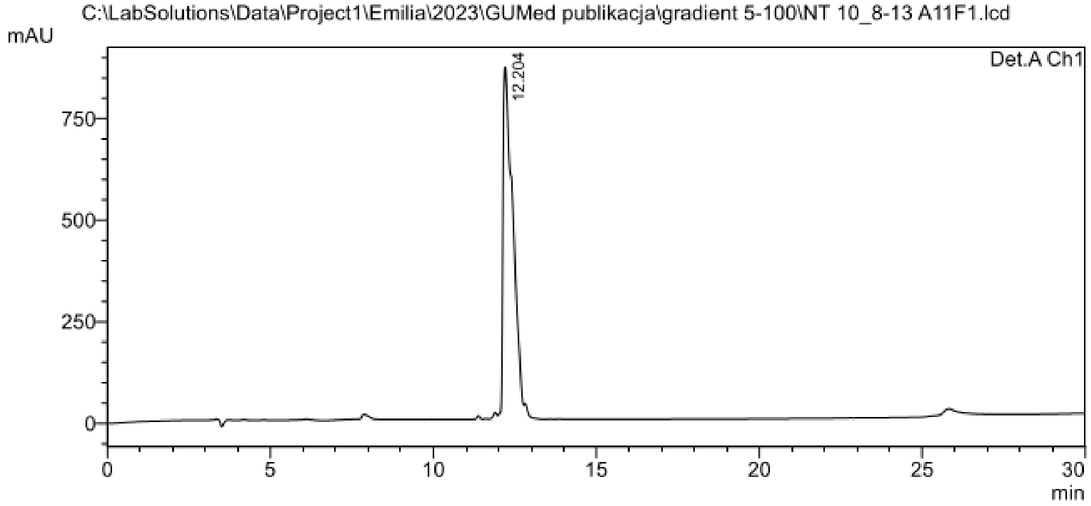

B.

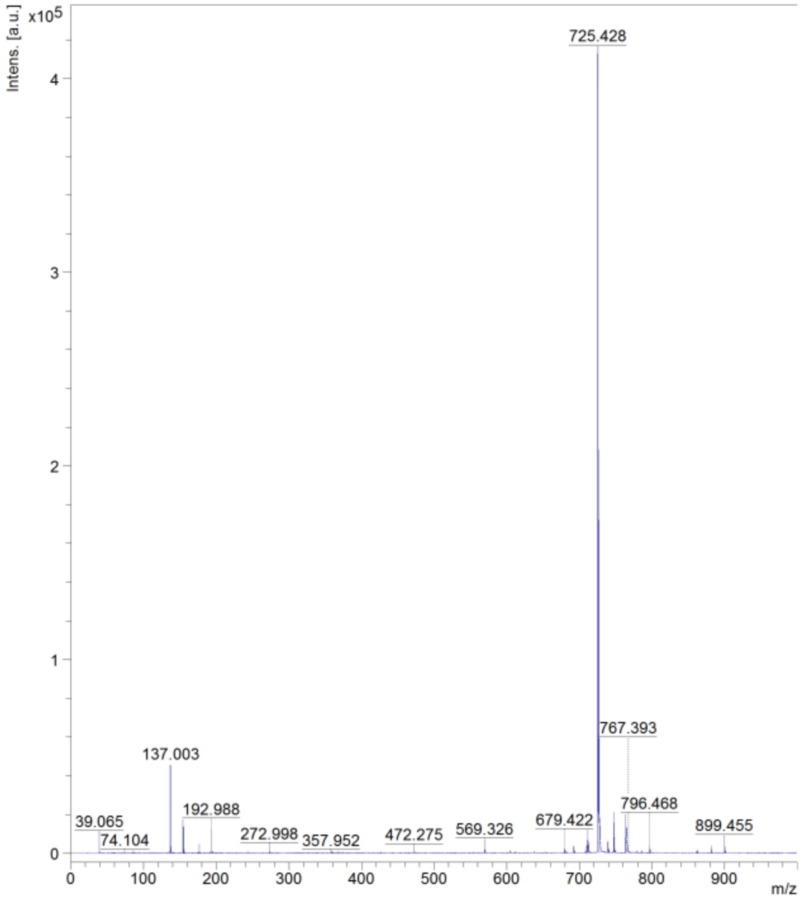

Figure. S12. (A) Chromatogram RP-HPLC with the tR 12.20 min and (B) mass spectra of the NT-10 peptide. Calculated mass: 724.91; found 725.43.

A.

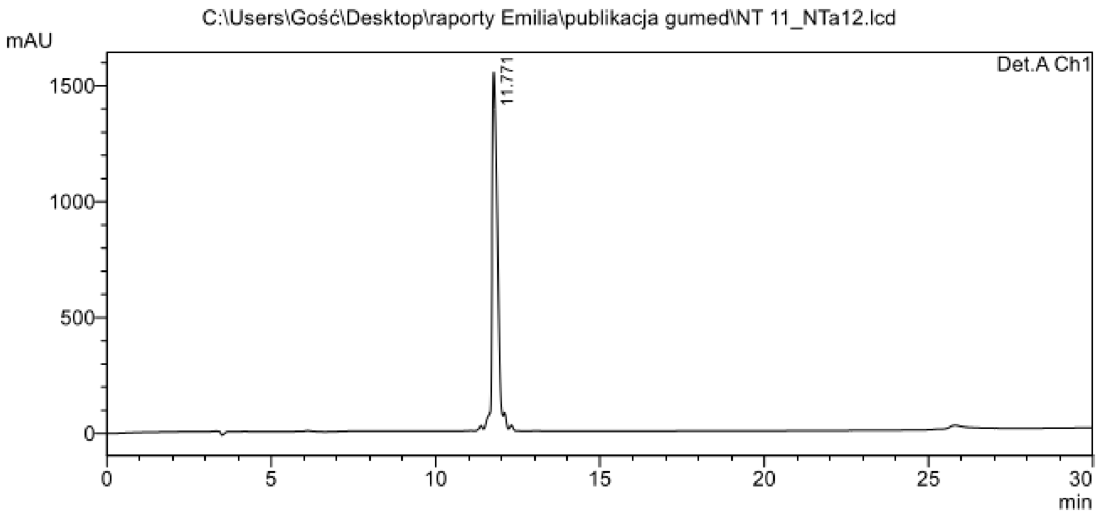

B.

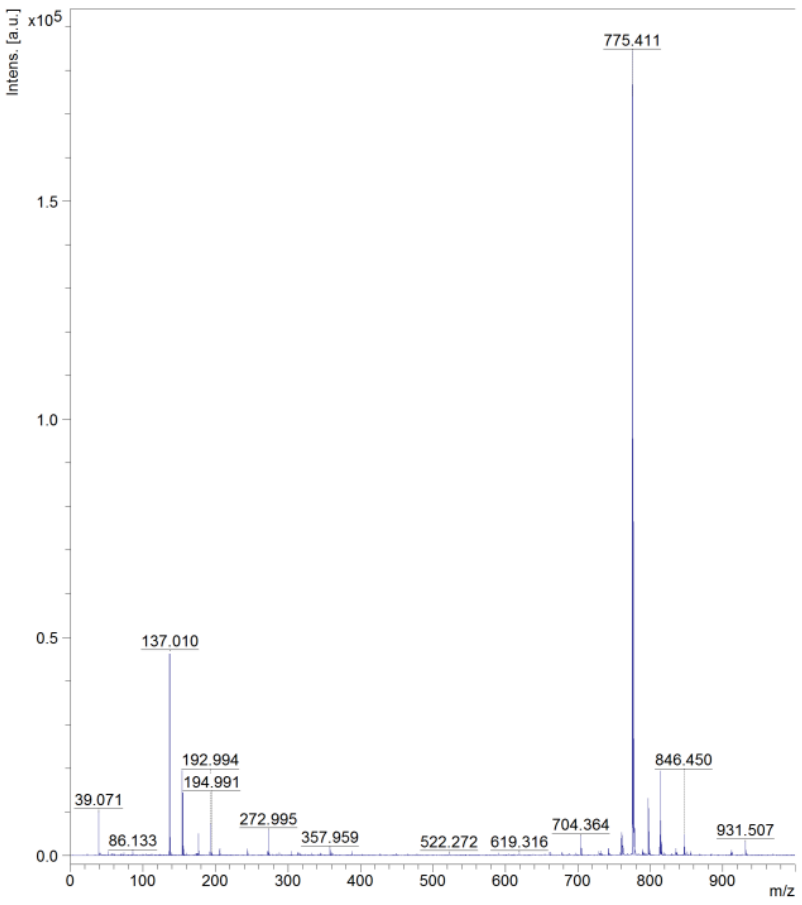

Figure. S13. (A) Chromatogram RP-HPLC with the tR 11.77 min and (B) mass spectra of the NT-11 peptide. Calculated mass: 774.98; found 775.41.

A.

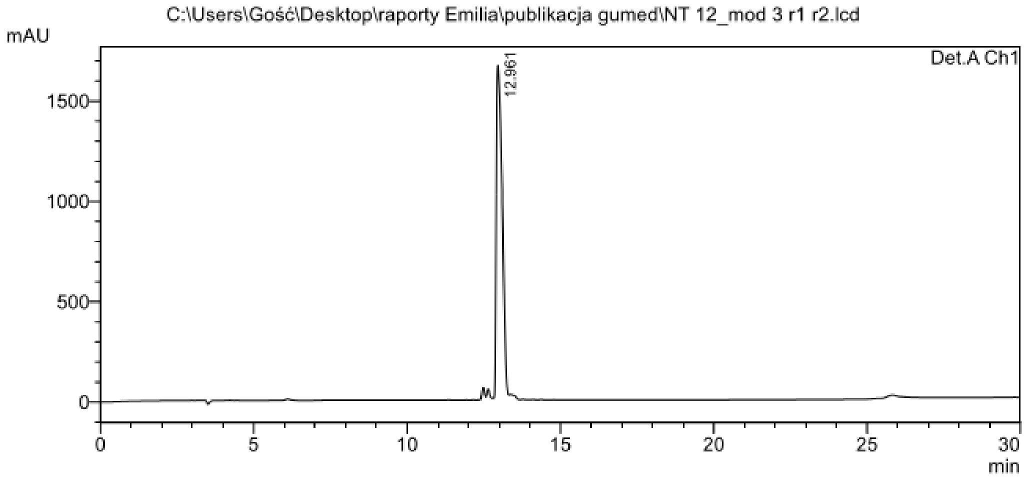

B.

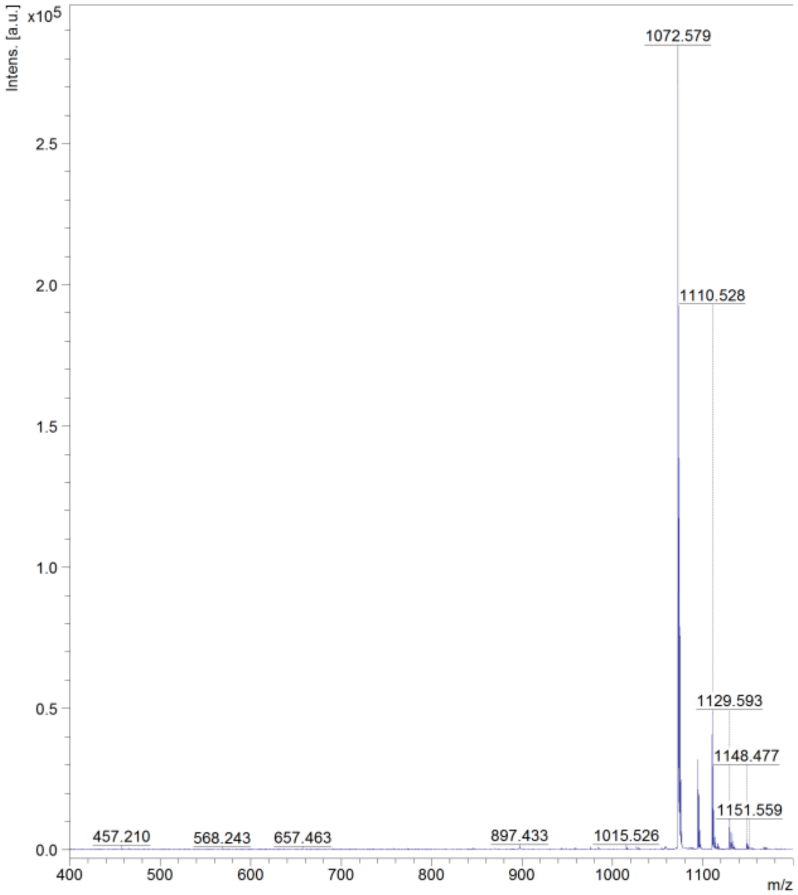

Figure. S14. (A) Chromatogram RP-HPLC with the tR 12.96 min and (B) mass spectra of the NT-12 peptide. Calculated mass: 1072.27; found 1072.58.

A.

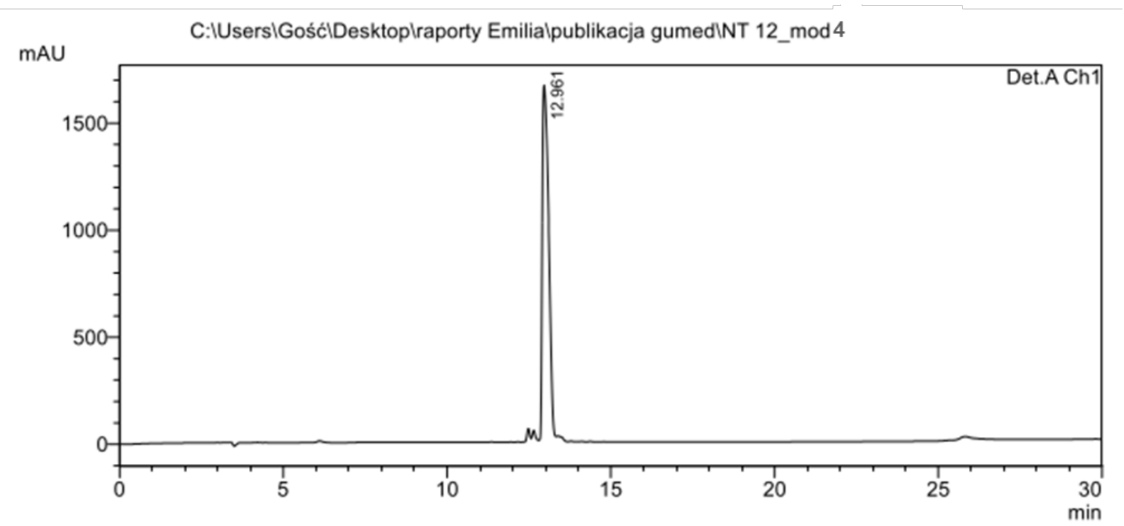

B.

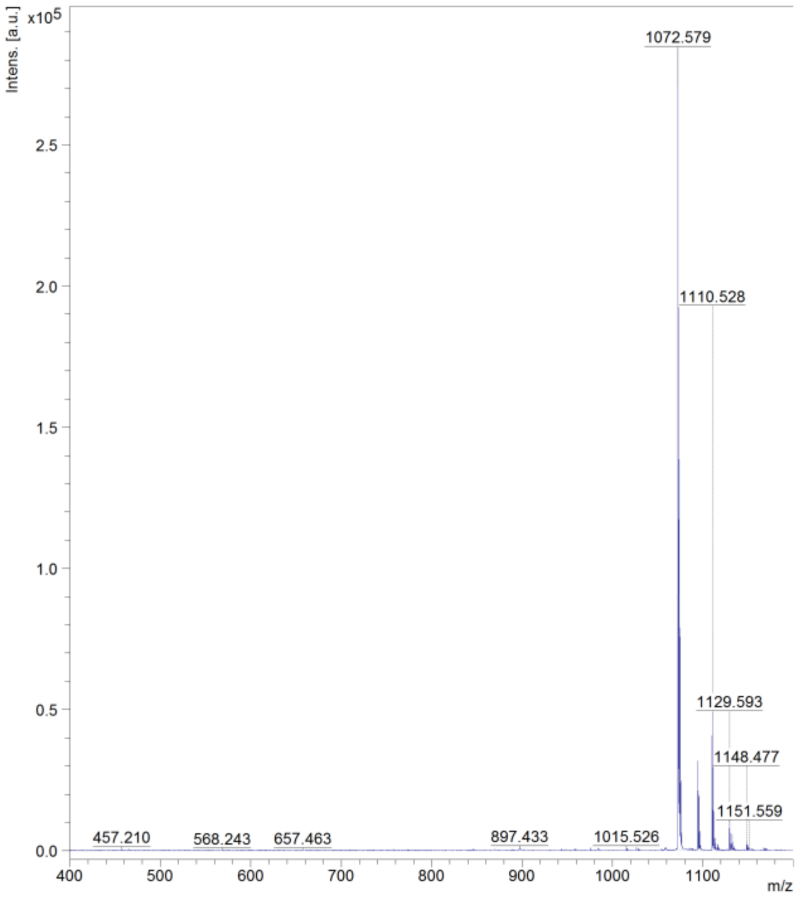

Figure. S15. (A) Chromatogram RP-HPLC with the tR 12.96 min and (B) mass spectra of the NT-13 peptide. Calculated mass: 1072.27; found 1072.56.

A.

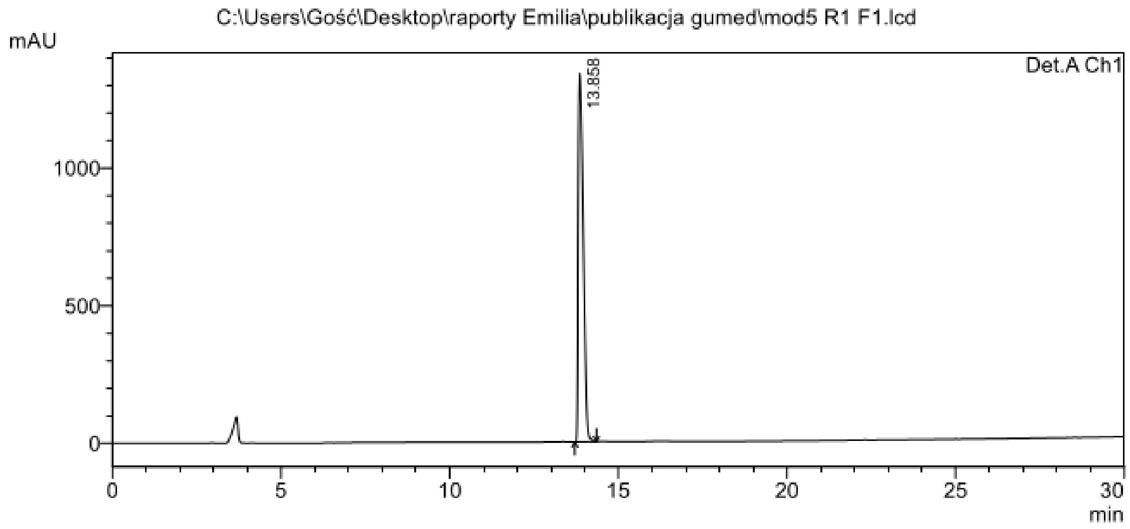

B.

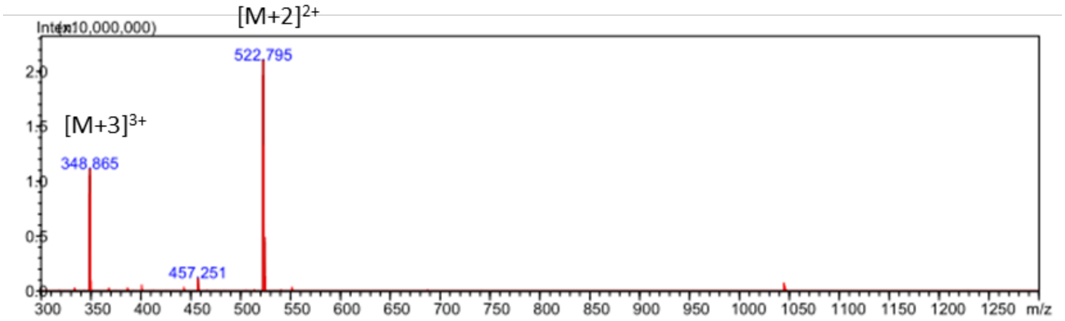

Figure. S16. (A) Chromatogram RP-HPLC with the tR 13.85 min and (B) ESI IT ToF mass spectra of the NT-14 peptide. Calculated mass: 1044.35; found 1044.58.

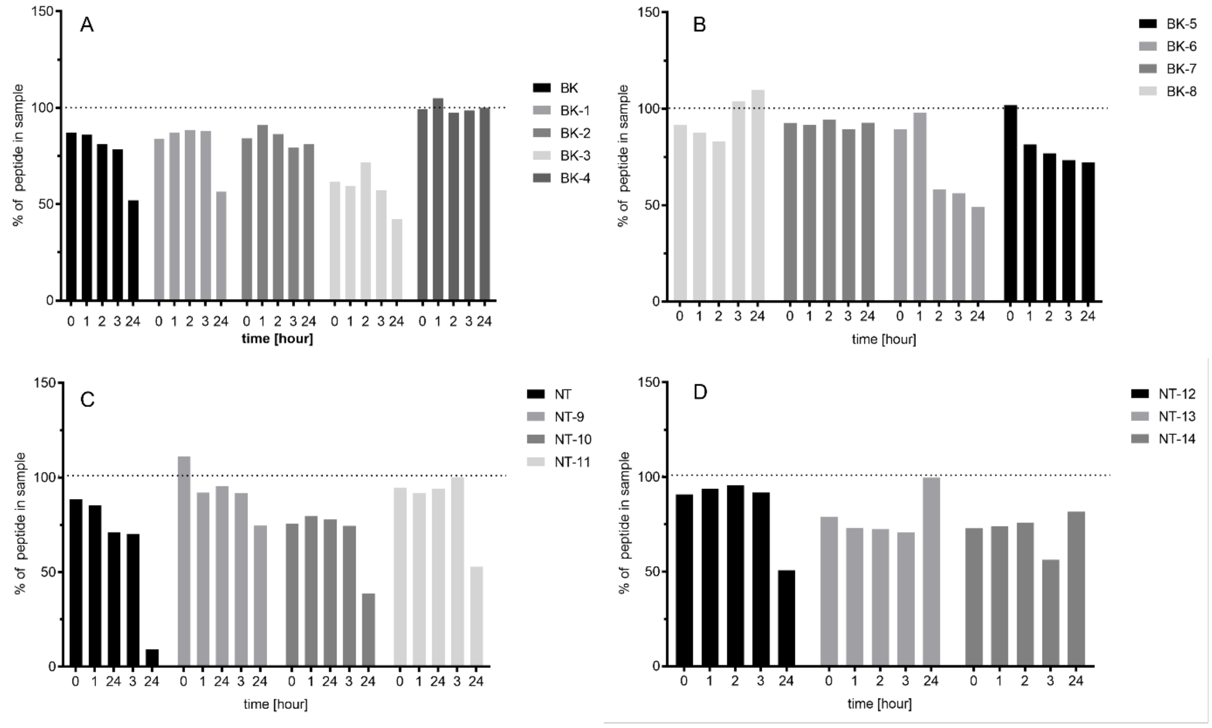

Figure. S17. Graph of the dependence of the decrease of the peptides concentration on incubation time with human plasma for: (**A)** analogues: BK, BK-1, BK-2, BK-3, BK-4; (**B**) analogues: BK-5, BK-6, BK-7, BK-8; (**C**) analogues: NT, NT-9, NT-10, NT-11; (**D**) analogues: NT-12, NT-13, NT-14.
